# Quercetin as a Natural Adjunct in Managing Plaque and Gingivitis in Adults: A Randomized Controlled Trial

**DOI:** 10.7759/cureus.109382

**Published:** 2026-05-21

**Authors:** Ankita Rawal, Kailash Asawa, Soumya S Das, Deep Gadhia, Diya Sharma, Priyadarshni Pathak

**Affiliations:** 1 Public Health Dentistry, Pacific Dental College and Hospital, Udaipur, IND

**Keywords:** antioxidant, chlorhexidine, gingivitis, placebo, plaque, quercetin

## Abstract

Background

Gingivitis, a reversible inflammatory condition of the gingiva, is primarily caused by plaque accumulation. Chlorhexidine is the gold standard for plaque control, but it is associated with side effects on prolonged use. Quercetin, a natural flavonoid with antioxidant and anti-inflammatory properties, has shown promise in vitro, but clinical evidence remains limited.

Aim

To compare the effectiveness of quercetin mouthwash with chlorhexidine and placebo in reducing dental plaque accumulation and gingivitis.

Materials and methods

A triple-blind, randomized controlled trial was conducted among 45 dental students aged 18-27 years, who were allocated to three groups: Quercetin (2%), chlorhexidine (0.2%), and placebo mouthwash. Plaque Index and Gingival Index were recorded at baseline, 15 days, and 30 days. Statistical analysis was performed using repeated measures two-way ANOVA with a significance level of p ≤ 0.05.

Results

Both quercetin and chlorhexidine groups demonstrated a statistically significant reduction in Gingival Index scores from baseline to 30 days (p < 0.001) compared to placebo. Plaque scores remained minimal in both groups, with no significant difference between quercetin and chlorhexidine at 15 and 30 days. Mild tooth staining was observed in the chlorhexidine group, while quercetin showed no adverse effects.

Conclusion

Quercetin mouthwash demonstrated efficacy alternative to chlorhexidine in reducing gingival inflammation and controlling plaque within the limits of this exploratory trial, without associated side effects, making it a promising natural alternative for long-term use. However, as formal non-inferiority testing was not performed, these findings should be interpreted as preliminary.

## Introduction

Antimicrobial mouthwashes serve as a crucial adjunct to prevent oral diseases, particularly in the prevention and management of periodontal disease, by effectively reducing dental plaque biofilm. Mouthwashes possess properties such as antiplaque action, tooth-whitening capability, and potential to control malodor (halitosis) [1].

Due to growing awareness of indigenous medical traditions, the use of herbal medicine has generated interest and led to the development of complementary and alternative therapies to promote healthcare in many parts of the world. This shift reflects a broader movement toward natural, culturally rooted alternatives that align with preventive health strategies. For a while now, herbal ingredients have been incorporated into oral care products, primarily in South Asian countries, to help people with gingivitis and maintain improved oral hygiene [2].

Essential oils from phytotherapeutic plants, which include active ingredients such as catechins, tannins, and sterols, were used to make herbal mouthwashes [3]. Flavonoids are secondary metabolites that are present in several kinds of plants and are vital to their survival because they carry out physiological functions and help the plant avoid negative environmental effects [4]. Quercetin (3, 3′, 4′, 5, 7- pentahydroxyflavone) is one of the most prevalent bioflavonoids present in fruits and vegetables, such as cherries, broccoli, red onion, and mango [3]. Its molecular structure is composed of two benzene rings, which are linked by a pyrone ring. Recently, quercetin has demonstrated a significant pharmacological value due to its antioxidant, anti-inflammatory, antibacterial, antineoplastic, anti-viral, anti-diabetic, neuroprotective, and anti-allergic properties [5]. The US Food and Drug Administration recently declared quercetin to be "generally recognized as safe" (GRAS) [3]. In light of these qualities, quercetin has gained wide acceptance and is used extensively in the medical field. 

Prior research has shown that quercetin has special antibacterial and anti-inflammatory properties against periodontitis [4]. In animal models of periodontitis, Cheng et al. (2010) observed that the quercetin group's mean bone resorption was considerably lower at eight and 12 days and suggested that it may improve periodontal destruction [6]. Quercetin showed effectiveness in combating *Porphyromonas gingivalis* and *Actinobacillus actinomycetemcomitans*; hence, it can aid in the treatment of periodontitis [7].

Gingivitis affects a large number of people worldwide and is regarded as an initial stage of periodontitis, which is a result of pathological alterations brought by the accumulated tooth surface plaque or biofilm [8]. Yang et al. (2017) demonstrated that the quercetin-doped adhesive (500 µg/mL) prevented the formation of *Streptococcus mutans* biofilm [9]. Vipin et al. (2019) demonstrated quercetin's anti-biofilm effectiveness against *Pseudomonas aeruginosa*, and the researchers found that at a dose of 500 µg/mL, quercetin totally inhibited (100%) all of the isolates tested in the research [10]. According to the findings of a study by Wang et al. (2018), quercetin inhibits the formation of biofilms by influencing the synthesis of sialic acid [11]. Inhibition of *Bacillus subtilis* in biofilm by quercetin was observed by Bordeleau et al. (2018), who reported that compared to the untreated control, 500 μg/mL of quercetin was adequate to reduce biofilm formation by 84% [12]. Quercetin was found to be 95% efficient in inhibiting the production of biofilms in *Enterococcus faecalis* [13].

The most commonly traded antibacterial mouthwash in the entire globe and the industry standard for mouthwash formulations is chlorhexidine. However, long-term usage of chlorhexidine has been linked to adverse side effects such as tooth discoloration, altered taste, and irritation of the oral mucosa. Alternative mouthwash formulations using natural components, such as different herbs, have received more attention. Herbal mouthwashes, including triphala, piper betel, and other ingredients, have demonstrated good plaque-reducing efficacy when compared with chlorhexidine [14]. The main goal of testing and researching so many mouthwashes is to find a better substitute for chlorhexidine.

Furthermore, despite promising in vitro and experimental evidence, limited human trials have evaluated the efficacy of quercetin in the management of plaque and gingivitis. Hence, the present study was conducted with the objective of determining the effectiveness of quercetin mouthwash in reducing dental plaque and gingivitis. The primary outcome measures were changes in Gingival Index and Plaque Index scores assessed using the Loe and Silness Gingival Index and Silness and Loe Plaque Index, respectively, to evaluate and compare the efficacy of quercetin and chlorhexidine mouthrinses on gingivitis and dental plaque accumulation. The secondary outcome measure was assessment of adverse effects associated with quercetin and chlorhexidine mouthrinses during the study period. The null hypothesis of the current research paper states that “There is no statistically significant difference in the effect of quercetin mouthwash, chlorhexidine mouthwash, and placebo mouthwash on dental plaque accumulation and gingival status among dental students at baseline, 15 days, and 30 days.”

## Materials and methods

Study design, study duration, study area, study population, and study setting

A prospective, triple-blind, concurrent parallel randomized controlled trial was conducted in three months (August 2025 to October 2025) to evaluate the effect of quercetin mouthwash, chlorhexidine mouthwash, and placebo mouthwash on dental plaque and gingival status among 45 dental students of Pacific Dental College and Hospital, Udaipur, Rajasthan. The study was conducted at the Department of Public Health Dentistry, Pacific Dental College and Hospital, a single-center academic and clinical setting, where recruitment and data collection were carried out among dental students.

Ethical clearance and official permission

The study protocol was reviewed by the Ethical Committee of Pacific Dental College and Hospital, Udaipur, and was granted ethical clearance (PDCH/25/EC-57) on June 7, 2025. An official permission was obtained from the Principal of Pacific Dental College and Hospital, Udaipur.

Trial registration

The registration number for this trial was CTRI/2025/07/090322, obtained on July 7, 2025.

Inclusion criteria and exclusion criteria

Subjects between 18 and 27 years of age who agreed to cooperate and were willing to participate in the study were included. Additionally, subjects with a Gingival Index greater than 1, a Periodontal Pocket Depth of 3 mm or less, and no Clinical Attachment loss were considered eligible. Subjects were excluded if they had been on antibiotic therapy or any mouthwash within the last two weeks, or had a history of hypersensitivity to chlorhexidine or any tested product used in the study. Those with a recent tooth extraction, any kind of oral lesions, or severe periodontal disease - characterized by purulent exudates, generalized mobility, and severe recession - were also excluded. Subjects who self-reported pregnancy, intent to become pregnant during the study, or were breastfeeding were not eligible, nor were those with severe crowding, malocclusion, or developmental disorders related to the oral cavity.

Sample size calculation

Sample size was calculated based on the study by Gunjal & Pateel (2024), with an expected mean difference of 2.281 and standard deviation of 2.563, at a power of 80% and level of significance at 5%, yielding a minimum sample of 12 participants per group [14]. Considering an attrition rate of 20%, the sample size was adjusted to 15 subjects per group, giving a total of 45 participants across three groups.

Informed consent

Before the investigation started, all subjects gave their written informed consent after being informed about the purpose and detailed procedure of the study.

Proforma details

A specially prepared format in the English language, exclusively designed for recording all the relevant data, was prepared. The proforma consisted of information regarding demographic details, Silness and Loe Plaque Index [15], and Loe and Silness Gingival Index [16] at baseline, 15 days, and 30 days, respectively.

Quercetin mouthwash

The formulation consisted of 2% quercetin, which functions as the principal active compound due to its antioxidant and anti-inflammatory properties. Sucrose was incorporated at a concentration of 0.3%, serving primarily as a stabilizing agent and potentially enhancing palatability in oral dosage forms. To facilitate the dispersion and solubilization of hydrophobic components, 0.01% sodium lauryl sulphate was included as a surfactant. Additionally, a preservative was added at a concentration of 0.001% to prevent microbial contamination and to ensure the overall stability and shelf life of the formulation.

Placebo mouthwash

The placebo formulation was prepared to match the test formulation in all aspects except for the active ingredient. It contained 0.3% Sucrose as a stabilizing and taste-enhancing agent, 0.01% sodium lauryl sulphate as a surfactant to aid in solubilization and dispersion of components, and 0.001% preservative to maintain microbiological stability. This composition ensured that any observed effects could be attributed specifically to the active compound, while the excipients remained consistent across both placebo and test formulations.

Training and calibration

All the examinations were carried out by a single examiner. Prior to the commencement of the study, the examiner was standardized and calibrated for Silness and Loe Plaque Index [15] and Loe and Silness Gingival Index [16] in the Department of Public Health Dentistry under an experienced epidemiologist. The examiner practiced the index first on 10 subjects. Then the examiner applied the criteria by examining a group of 10 subjects twice on successive days. The intra-examiner reliability was assessed using Cohen's Kappa statistic, yielding a Kappa value of 0.90 (95% CI: 0.82-0.98), indicating almost perfect agreement, confirming acceptable examiner consistency prior to commencement of the study.

Random allocation of the test products and instructions

A total of 45 participants were randomly assigned to one of the three different groups (n=15 each) in a 1:1:1 ratio using simple randomization by a lottery method. The random allocation sequence was prepared by an independent person uninvolved in the study, who placed equal numbers of coded slips in a sealed opaque box. The principal investigator enrolled participants after verifying eligibility, and each participant drew a slip to determine group assignment: Group I included quercetin mouthwash, Group II included chlorhexidine mouthwash, and Group III included placebo mouthwash. A blinded research assistant assigned the corresponding coded mouthwash to each participant. The clinician who performed all measurements was blinded to the treatment arms of the subjects. Also, the subjects and evaluator were blinded, leading to a triple blind study design. The randomization codes were not broken until data had been collected.

**Figure 1:**
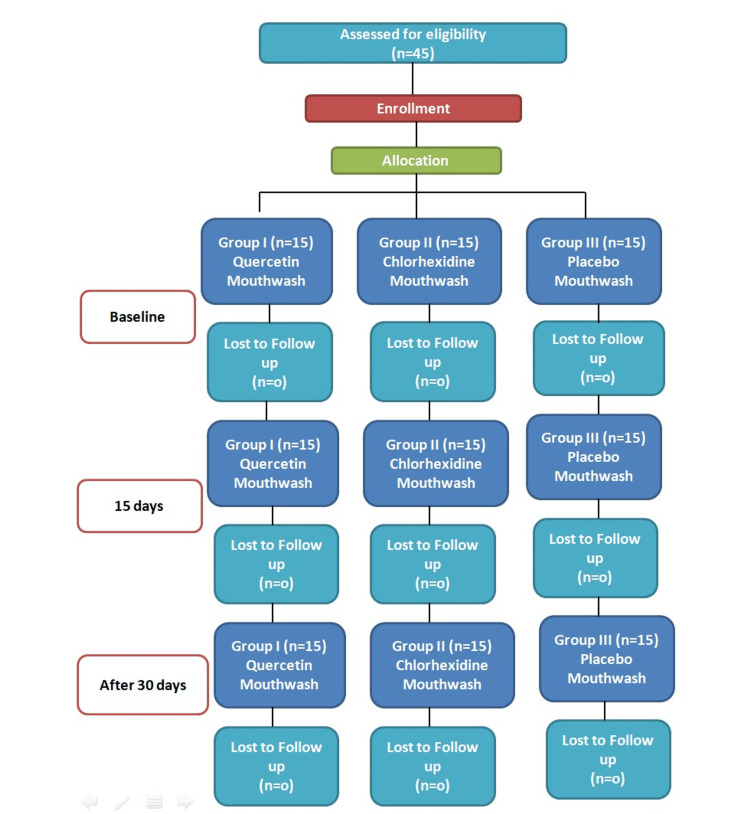
CONSORT flow diagram showing participant enrollment, random allocation, follow-up, and analysis between groups. "n" is the number of study participants. CONSORT, Consolidated Standards of Reporting Trials.

Data were analyzed on an intention-to-treat (ITT) basis, whereby all 45 randomized participants were included in the analysis according to their originally assigned group, regardless of compliance or protocol deviations. No participants were excluded after randomization (as shown in the CONSORT flow diagram, Figure 1). This approach was adopted to preserve the integrity of randomization and minimize the risk of attrition bias. Primary and sensitivity analyses were therefore conducted on the complete dataset of 45 participants.

Methodology

Prior to the commencement of the study, all participants underwent oral prophylaxis and polishing. A standardized tooth brushing technique was demonstrated by the investigator to ensure that participants clearly understood the brushing instructions. All subjects were provided with Colgate Regular toothpaste and a Colgate soft manual toothbrush (Colgate-Palmolive Company, New York, NY, USA) to brush twice a day. After two weeks (washout period), subjects reported to the Department of Public Health Dentistry and were randomly allocated to three groups of 15 participants each. Each group was randomly allocated to one of the three mouth rinses. Prior to the start of the study, Plaque and Gingival Index were recorded at the baseline. All products were blinded to the subjects and the examiner by way of a wrap. Participants in all three groups were instructed to rinse their mouth with 10 ml of mouth rinse twice daily before breakfast and after dinner for a period of three minutes.

The participants were asked to follow the given instructions regarding the oral hygiene practices and rinsing procedure. Participants were recalled, and the Plaque and Gingival Index were recorded at 15 days and 30 days from the baseline. Baseline Plaque Index scores of 0.00 ± 0.00 across all groups resulted from the oral prophylaxis and polishing performed prior to baseline recording, intentionally standardizing oral hygiene status to eliminate pre-existing variability. The study protocol and statistical analysis plan are available from the corresponding author upon reasonable request.

Statistical analysis

The recorded data were compiled and entered into a spreadsheet computer program, then exported to the data editor page in IBM SPSS Statistics, Version 30 (IBM Corp., Armonk, NY, USA). Baseline demographic and clinical characteristics, including age, sex, Plaque Index, and Gingival Index, were tabulated and stratified by intervention group; between-group differences were evaluated using one-way ANOVA for continuous variables and the Chi-square test for categorical variables. Prior to analysis, normality was assessed using the Shapiro-Wilk test, sphericity using Mauchly's test with Greenhouse-Geisser correction where violated, and homogeneity of variance using Levene's test. Repeated-measures two-way analysis of variance followed by a Bonferroni post-hoc test was used to compare the effectiveness of the mouth rinses between three groups at baseline, 15-day, and 30-day intervals. The model examined the Time × Group interaction as the primary effect of interest, with time (baseline, 15 days, 30 days) and group (quercetin, chlorhexidine, placebo) as within- and between-subject factors, respectively. The primary pre-specified comparison was between Group I (quercetin) and Group II (chlorhexidine) at 30 days, with comparisons against placebo considered exploratory. Test statistics (F-value) along with corresponding p-values and 95% confidence intervals for between-group mean differences were calculated and reported. Effect sizes were reported as partial eta-squared (η²) for repeated-measures ANOVA and Cohen's d for pairwise comparisons. Where between-group differences were non-significant, results are reported descriptively without equivalence claims. Confidence level and level of significance were set at 95% and 5%, respectively (p ≤ 0.05).

## Results

In the present study, a 30-day follow-up was conducted to assess and compare the effect of quercetin and chlorhexidine mouth rinse on dental Plaque and Gingival Index scores. Prior to analysis, the Shapiro-Wilk test confirmed normality, the Mauchly's test confirmed sphericity, and Levene's test confirmed homogeneity of variance across all groups (p>0.05). 

Table 1 shows the demographic distribution across the study population. The majority of participants were female (n=39, 86.7%), while males constituted a smaller proportion (n=6, 13.3%). Regarding age distribution, the majority of participants were in the 22-23 year age group (n=30, 66.7%), followed by those aged 24-25 years (n=13, 28.9%). Only a small proportion of participants belonged to the 26-27 year age group (n=2, 4.4%).

**Table 1:** Distribution of demographic variables across the study population. Data are expressed as frequency (n) and percentage (%). Percentages are calculated based on total sample size (n = 45). No inferential statistical test was applied as the data are descriptive in nature.

Variables		n (%)
Gender	Male	6 (13.3%)
	Female	39 (86.7%)
Age (in years)	22-23	30 (66.7%)
	24-25	13 (28.9%)
	26-27	2 (4.4%)

Table 2 reveals the comparative assessment of mean Gingival Index scores among all three groups, including quercetin, chlorhexidine, and placebo at baseline, after 15 days, and after 30 days. At baseline, the mean gingival score was comparable among the quercetin group (1.48±0.18), the chlorhexidine group (1.44±0.16), and the placebo group (1.31±0.19). No statistically significant between-group differences were observed at baseline for the Gingival Index (p>0.05) or Plaque Index (p>0.05), confirming successful randomization. After 15 days of intervention, both quercetin (Mean Difference=0.24) and chlorhexidine (Mean Difference=0.21) groups revealed a significant reduction in gingival score (p<0.001), while the placebo group (Mean Difference=0.04) exhibited a slight increase in mean gingival score, which was statistically significant (p<0.001). After 30 days, the quercetin group demonstrated a significant reduction in mean gingival score (Mean Difference=0.21 (p<0.001)). Also, chlorhexidine demonstrated a slight reduction in mean gingival score (Mean Difference=0.17 (p<0.001)), and the placebo group showed a significant increase in the same (Mean Difference=0.02 (p<0.001)). There was no significant difference in mean gingival scores after 15 days and after 30 days between the quercetin and chlorhexidine groups. However, the placebo group showed significantly higher mean gingival scores than both the quercetin and chlorhexidine groups after 15 days and 30 days of intervention. The F-value for the study indicated a significant effect of time (F=58.73), mouthwash group (F=9.38), and interaction effect (F=50.10), reflecting significant variability in Gingival Index changes across time intervals and among the different mouthwash groups. These findings suggest that both quercetin and chlorhexidine were effective in reducing gingival inflammation, whereas the placebo group exhibited an increase in Gingival Index scores.

**Table 2:** Comparative assessment of Gingival Index among study groups at different time intervals. Descriptive statistics are expressed as Mean ± Standard Deviation (SD). A repeated measures two-way analysis of variance (ANOVA) followed by a Bonferroni post-hoc test was performed to evaluate differences between mouthwash groups over time. F-values indicate between-group variability. Statistical significance was set at p≤0.05. ^*^ indicates statistically significant differences. ^a, b, c, d, e^ indicates statistically significant differences between mouthwash groups at different time intervals.

Mouthwash	Gingival Index (Mean±SD)			p-value	F-value
	Baseline	After 15 days	After 30 days		
Quercetin	1.48±0.18^a^	1.24±0.27^b^	1.03±0.28^c^	<0.001^*^	89.46
Chlorhexidine	1.44±0.16^a^	1.23±0.25^b^	1.06±0.26^c^	<0.001^*^	
Placebo	1.31±0.19^a^	1.35±0.24^d^	1.37±0.25^e^	<0.001^*^	
p-value	0.03^*^	<0.001^*^	<0.001^*^	<0.001^*^ (Interaction effect)	
F-value	9.38^*^				75.65 (Interaction effect)

Table 3 illustrates the comparative assessment of mean plaque scores among all three groups, including quercetin, chlorhexidine, and placebo at baseline, after 15 days, and after 30 days. At baseline, the mean plaque scores were similar among the quercetin group (0.00±0.00), the chlorhexidine group (0.00±0.00), and the placebo group (0.00±0.00). After 15 days of intervention, the quercetin (Mean Difference= 0.01) and chlorhexidine (Mean Difference= 0.04) groups showed a minimal increase in plaque scores, but the placebo group (Mean Difference= 0.09) showed a significant increase in plaque score (p<0.001). After 30 days, the quercetin group (Mean Difference= 0.04) and chlorhexidine group (Mean Difference= 0.06) demonstrated a slight increase in mean plaque scores, which was non-significant, but the placebo group (Mean Difference= 0.12) showed a significant increase in mean plaque scores (p<0.001). At 15 days, the comparison between quercetin and chlorhexidine revealed a negligible mean difference in plaque scores (Mean Difference = 0.03), which was not significant, indicating that both agents produced a similar reduction in plaque accumulation. When compared to the placebo group, both quercetin (Mean Difference = 0.08) and chlorhexidine (Mean Difference = 0.05) demonstrated significantly lower mean Plaque Index scores. After 30 days, the difference between quercetin and chlorhexidine remained small (Mean Difference = 0.05), which was statistically non-significant, supporting the comparable efficacy of the two treatments over time. However, both agents exhibited more pronounced statistically significant differences when compared to the placebo. The F-values for interaction effects were 105.84 and 14.79, respectively, demonstrating a high degree of variability in plaque score changes among the groups as well as a statistically significant difference in the pattern of change across time intervals.

**Table 3:** Comparative assessment of Plaque Index among study groups at different time intervals. Descriptive statistics are expressed as Mean ± Standard Deviation (SD). A repeated measures two-way analysis of variance (ANOVA) followed by a Bonferroni post-hoc test was performed to evaluate differences between mouthwash groups over time. F-values indicate between-group variability. Statistical significance was set at p≤0.05. ^*^ indicates statistically significant differences. ^a, b, c, d, e^ indicates statistically significant differences between mouthwash groups at different time intervals.

Mouthwash	Plaque Index (Mean±SD)			p-value	F-value
	Baseline	After 15 days	After 30 days		
Quercetin	0.00±0.00^a^	0.01±0.03^b^	0.05±0.02^c^	<0.001^*^	28.36
Chlorhexidine	0.00±0.00^a^	0.04±0.03^b^	0.10±0.03^c^	<0.001^*^	
Placebo	0.00±0.00^a^	0.09±0.08^d^	0.21±0.08^e^	<0.001^*^	
p-value	-	<0.001^*^	<0.001^*^	<0.001^*^ (Interaction Effect)	
F-value	105.84				14.79 (Interaction Effect)

Any harms or unintended effects in each group

Light brownish stain observed in four out of 15 participants of the chlorhexidine group after 30-days of post-rinse. However, no staining was seen in any participants of the quercetin group. Participants with stained teeth were immediately followed by complete oral prophylaxis, adjunct with polishing of teeth.

## Discussion

Mouthwashes are considered a supplement to oral cleanliness and are utilized in the conveyance of dynamic agents to the teeth and gums since they can repress bacterial colonization growth, and furthermore interfere with the development of biofilm [17]. Natural herbal products have always been an integral part of Indian history for combating a variety of diseases. Quercetin is a flavonoid that has been the subject of extensive research owing to its diverse pharmacological activities, including antioxidant, antiviral, immune-modulatory, and anticancer activity with a low toxicity profile [5]. Antioxidants play an imperative part in the control of gingival irritation by hindering oxidative stress. This clinical trial was designed to assess the effectiveness of quercetin and chlorhexidine mouthrinses compared to a placebo in reducing gingival inflammation and dental plaque accumulation among the adult population. The present research was a triple-blind study wherein the investigator, study subjects, as well as the evaluator, were not aware of which group the subjects belonged to, and coding was done for each group and individual. The result of this exploration indicated that before any intervention, there were no significant differences in the baseline values between the three groups. So, it was conceivable to make a comparison between the viability of the three diverse mouth rinses on the gingival and dental plaque scores. No symptoms or mishappenings were seen during the research procedure.

In the present research, after rinsing with respective mouthwashes, the results of the trial demonstrated that there were statistically lower mean Plaque Index scores obtained in the quercetin group than the negative control, that is, the placebo, and comparable plaque reduction scores with the positive control, that is, chlorhexidine mouthrinse at 15 days and 30 days. Thus, within the limits of this exploratory trial, quercetin mouthwash demonstrated comparable efficacy in inhibiting plaque to chlorhexidine, though formal non-inferiority testing was not performed. Nonetheless, the current study provides preliminary clinical evidence suggesting that quercetin mouthwash may serve as a natural and biocompatible alternative to conventional chemical agents like chlorhexidine, demonstrating comparable efficacy in plaque reduction within the limits of this exploratory trial. The comparable clinical efficacy of quercetin can be attributed to its strong antioxidant, anti-inflammatory, and antimicrobial properties, which help inhibit bacterial colonization and modulate inflammatory responses in gingival tissues. These findings align with previous in vitro and animal studies that have demonstrated quercetin’s antibacterial activity against key oral pathogens and its role in reducing periodontal destruction.

Some vivo studies showed that quercetin reduced alveolar bone loss in periodontitis and enhanced the durability of dental adhesives by inhibiting inflammation, bacterial growth, and matrix metalloproteinase (MMP) activity (Napimoga et al., 2009) [4]. Quercetin has shown effects on mineralized dental tissues by promoting enamel remineralization, enhancing resistance to caries, and improving the adhesion and durability of restorative materials (Cheng et al., 2010) [6]. Its anti-inflammatory and antioxidant properties help preserve dentin structure, while its combination with collagen reinforces dentin’s mechanical strength (Hong et al., 2023) [18].

Quercetin exhibits a broad spectrum of therapeutic effects, including antioxidant, anti-inflammatory, antimicrobial, antiviral, antihypertensive, anticancer, and antidiabetic effects, on overall health; it also has wound-healing properties [19]. In this in vivo study, both quercetin and chlorhexidine significantly reduced Plaque Index score compared to placebo, which aligns with in vitro findings where quercetin mouthwash formulations demonstrated broad-spectrum bactericidal activity comparable to commercial herbal mouth rinses (Akshayaa et al., 2024) [3]. Contextualizing these findings within the broader herbal mouthwash literature, trials evaluating triphala and propolis mouthwashes have similarly reported significant reductions in plaque and Gingival Index scores compared to placebo, with effect sizes broadly comparable to those observed in the present study, suggesting that quercetin represents a clinically relevant addition to the spectrum of herbal alternatives available for gingivitis management. Furthermore, Geoghegan et al. (2008) reported no significant difference between quercetin and chlorhexidine after six hours of incubation, supporting the comparable antimicrobial potential observed in the present trial [20]. The mechanism of gingival improvement may also involve Quercetin’s anti-inflammatory activity, including modulation of pro-inflammatory cytokines and oxidative stress pathways (Mooney et al., 2021) [21]. In animal models of periodontitis, Cheng et al. (2010) observed that the quercetin group's mean bone resorption was considerably lower at eight and 12 days and suggested that it may improve periodontal destruction [6]. However, there remains a lack of clinical evidence on the effect of quercetin on oral microbiome composition and long-term gingival health, indicating the need for further clinical trials.

Quercetin showed effectiveness in combating *Porphyromonas gingivalis* and *Actinobacillus actinomycetemcomitans*, hence it may help in the treatment of periodontitis. Gingivitis is an inflammatory condition of the gingival tissues primarily caused by bacteria in plaque, including *Streptococcus*, *Fusobacterium*, *Actinomyces*, *Veillonella*, and *Treponema *[20]. Of these, *Treponema denticola* has shown strong evidence for its association with gingival disease pathogenesis. Flavonoids like quercetin possess antibacterial, antioxidant, and anti-inflammatory properties, which could explain their therapeutic benefits. Previous studies have demonstrated that quercetin inhibits the growth of oral pathogens, including *Streptococcus mutans*, *Streptococcus sobrinus*, *Lactobacillus acidophilus*, *Actinobacillus actinomycetemcomitans*, and *Prevotella intermedia*. These effects may be due to quercetin's ability, which interferes with bacterial metabolic pathways, such as inhibition of thymidylate synthase, thereby disrupting the replication of microbes.

One notable advantage observed in the present study was the absence of adverse effects related to chlorhexidine, such as tooth staining, altered taste, and mucosal irritation, in the quercetin group. This finding supports the clinical benefit of quercetin as a safer alternative to chlorhexidine for long-term use. James et al. (2017) documented that chlorhexidine commonly induces side effects such as taste disturbance, mucosal soreness, and staining, which limit patient compliance [22].

The limited availability of human clinical studies on quercetin mouthrinse represents an important gap in the literature. While previous data and in vitro studies are promising, large-scale randomized controlled trials with extended follow-up periods are needed to confirm these findings and clarify the underlying mechanisms. Additionally, subject-reported outcomes, such as satisfaction and tolerance, should be evaluated in future research to assess compliance and acceptability compared to chlorhexidine mouthrinse.

The strength of this study lies in its controlled design and the direct comparison between quercetin, chlorhexidine, and placebo mouth rinses. However, limitations include the relatively short duration of follow-up, which is 15 days and one month, and the absence of microbiological analysis to confirm changes in harmful bacteria. Incorporating such assessments, along with biomarkers of inflammation, would provide a more comprehensive understanding of the quercetin therapeutic role for managing gingivitis. Consequently, doing further examinations in this field will give more information and will end up being useful in studying the impacts of different herbal restorative plants on periodontal maladies.

Limitations

The present study had certain limitations. The duration of follow-up was relatively short, which may not adequately reflect the long-term effectiveness of quercetin mouthwash on gingival health and plaque control. Microbiological analysis was not performed to evaluate changes in specific oral pathogens, thereby limiting insight into the antimicrobial mechanism of quercetin. In addition, biochemical and inflammatory markers were not assessed, which could have provided a better understanding of its anti-inflammatory effects. Additionally, the trial was restricted to a small sample size, which may limit the generalizability of the findings to the broader population and increase the risk of type II error. Although a triple-blind design was adopted, the possibility of residual performance or detection bias cannot be completely excluded. Furthermore, as this was a superiority trial and not designed as a non-inferiority study, the absence of a statistically significant difference between quercetin and chlorhexidine groups should not be interpreted as evidence of equivalence, and formal non-inferiority conclusions cannot be drawn.

## Conclusions

The present study provides clinical evidence supporting the comparable efficacy of quercetin mouthwash as a potential alternative to chlorhexidine, within the limits of this exploratory trial for controlling gingival inflammation and plaque accumulation. Both mouth rinses demonstrated significant reductions in Gingival Index scores and maintained minimal plaque levels over a 30-day period. Importantly, while chlorhexidine remains effective, its long-term use is limited by undesirable side effects, such as tooth staining and altered taste, as observed in a subset of participants in this study. In contrast, quercetin exhibited no adverse effects, making it a safer and more acceptable option for prolonged use. It should be noted, however, that formal non-inferiority testing was not conducted in this trial; therefore, findings of comparable efficacy should be interpreted as preliminary rather than confirmatory.

Despite promising results, the study acknowledges certain limitations, including a relatively short duration of follow-up and the absence of microbiological and biochemical analyses to confirm changes at the microbial and cellular levels. Future research should incorporate long-term follow-ups, larger and more diverse populations, and comprehensive microbiological evaluations to further establish quercetin’s role in oral healthcare. In conclusion, quercetin mouthwash represents a safe, promising, and biocompatible herbal alternative to chlorhexidine for managing gingivitis and maintaining oral health. Its integration into preventive dentistry protocols could significantly reduce reliance on synthetic agents while enhancing patient compliance and safety.
